# The Emerging Roles of Rad51 in Cancer and Its Potential as a Therapeutic Target

**DOI:** 10.3389/fonc.2022.935593

**Published:** 2022-07-07

**Authors:** Ziyi Wang, Renxiang Jia, Linlin Wang, Qiwei Yang, Xiaohai Hu, Qiang Fu, Xinyu Zhang, Wenya Li, Yi Ren

**Affiliations:** ^1^ Department of Thoracic Surgery, Shenyang Chest Hospital & Tenth People’s Hospital, Shenyang, China; ^2^ Department of Thoracic Surgery, First Affiliated Hospital of China Medical University, Shenyang, China

**Keywords:** DNA repair, PARP inhibitor, Rad51, therapy resistance, prognosis

## Abstract

Defects in DNA repair pathways are emerging hallmarks of cancer. Accurate DNA repairs and replications are essential for genomic stability. Cancer cells require residual DNA repair capabilities to repair the damage from replication stress and genotoxic anti-tumor agents. Defective DNA repair also promotes the accumulation of genomic changes that eventually lead to tumorigenesis, tumor progression, and therapeutic resistance to DNA-damaging anti-tumor agents. Rad51 recombinase is a critical effector of homologous recombination, which is an essential DNA repair mechanism for double-strand breaks. Rad51 has been found to be upregulated in many malignant solid tumors, and is correlated with poor prognosis. In multiple tumor types, Rad51 is critical for tumor metabolism, metastasis and drug resistance. Herein, we initially introduced the structure, expression pattern of Rad51 and key Rad51 mediators involved in homologous recombination. Additionally, we primarily discussed the role of Rad51 in tumor metabolism, metastasis, resistance to chemotherapeutic agents and poly-ADP ribose polymerase inhibitors.

## Introduction

Defects in DNA repair pathways are hallmarks of cancer. Cancer cells require residual DNA repair abilities to repair the damage from replication stress and genotoxic anti-tumor agents (1). Therefore, defective DNA repair often leads to genomic instability. Defective DNA repair also promotes the accumulation of genomic changes that eventually lead to tumorigenesis, tumor progression, and therapeutic resistance to DNA-damaging anticancer therapy (2). To date, various inhibitors of DNA damage response (DDR) are in preclinical and clinical development, making DDR pathways ideal targets for therapeutic intervention (3).

**Figure 1:**
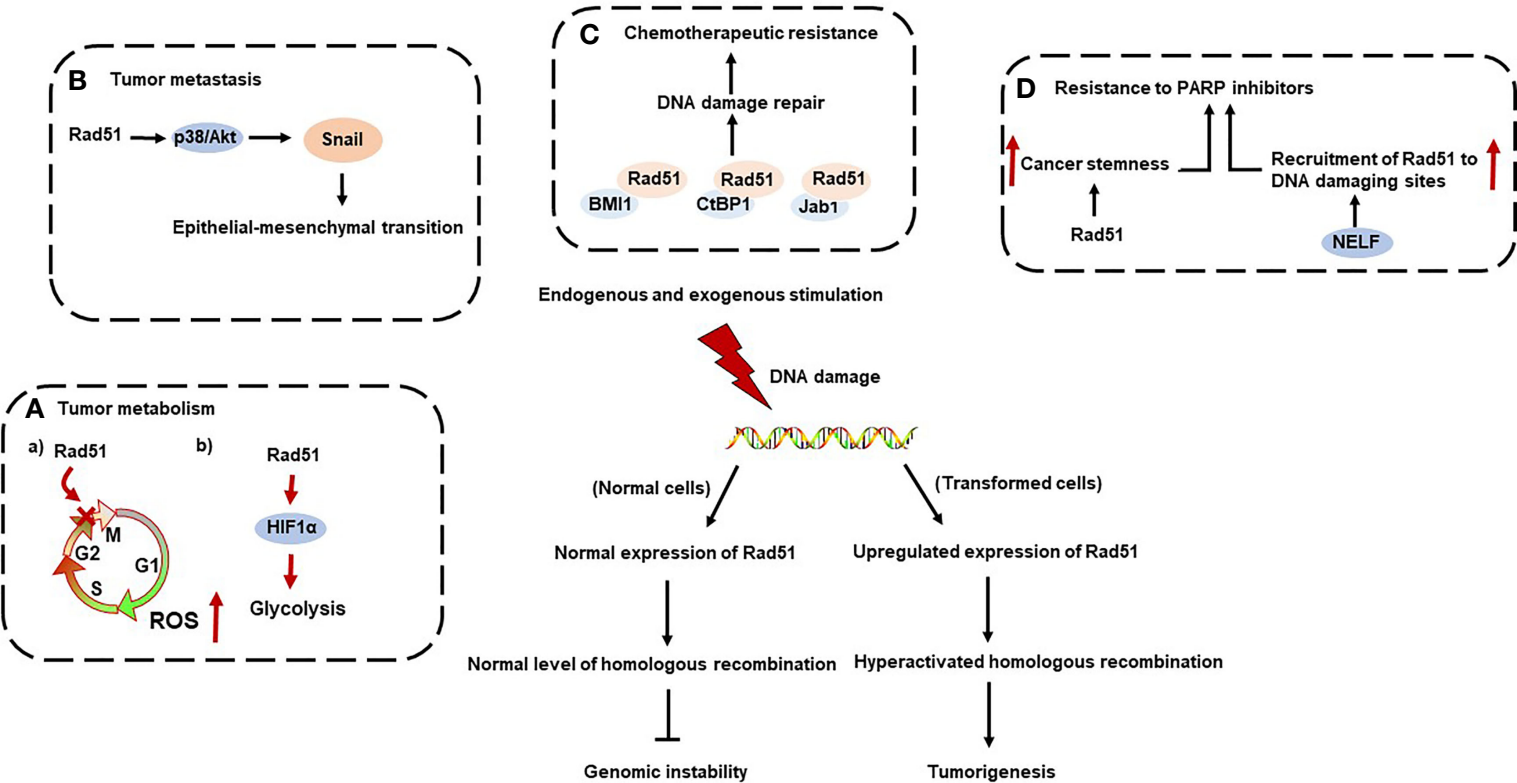
Mechanisms of Rad51 in tumor initiation and progression.

Rad51 is a DNA-binding protein, which can regulate nucleases, helicases, DNA translocases, and signaling proteins to function as a regulator of replication stress, such as mediating fork reversal and restoring repaired forks (4). In multiple cancer models, increased Rad51 expression is associated with poor clinical outcomes and adverse clinicopathological features (5–7). Rad51 is also engaged in the tumor initiation and development in multiple cancer types (8–10). Herein, we initially introduced the structure, expression pattern of Rad51 and key Rad51 mediators involved in homologous recombination response (HRR). Additionally, we primarily discussed the role of rad51 in tumor metabolism, metastasis, resistance to chemotherapy and poly-ADP ribose polymerase (PARP) inhibition (**Figure 1**).

## Rad51 Structure and Expression Pattern

The key role of Rad51 in DNA repair has sparked a wide variety of investigations on its structure. Rad51 contains an ATPase core domain similar to those present in helicases that hydrolyze nucleotide triphosphates (11). This domain includes a Walker A motif and a Walker B motif, which mediate ATP binding and hydrolysis. The presynaptic and postsynaptic complexes of Rad51 are essential for its function. ATP binds to Rad51 and activates the formation of the presynaptic filament, which assembles the recombinase promoters into a filament on single-stranded DNA. Next, strand exchange and joint molecule formation happens within the postsynaptic complex (12). These filaments are important for homology search and invading strand extension of a homologous template, which result in homology-mediated repair.

The regulation of Rad51 expression is complex and dynamic in tumor cells, as illustrated in **Table 1**. Transcription factors engaged in Rad51 expression is crucial for the response of tumor cells to DNA-damaging agents. It has been well established that CDK12/CDK13, E2F1 and FOXM1 can directly bind to the promoter of Rad51 to transactivate Rad51 expression (13). Other transcriptional factors such as E2F7, E2F4 and p53 exert its gene regulatory function as transcription repressors of Rad51 to inactivate Rad51 expression. Recent study also found that high mobility group A1 (HMGA1) can directly bind to A/T-rich DNA sequences located in the promoter regions of Rad51 and transcriptionally activate its expression to mediate irradiation resistance of cholangiocarcinoma cells (14; 15). Post-translational regulation of Rad51 participates in the activation or stabilization of Rad51 protein, which may provide novel anti-tumor targets for cancer management. A de-ubiquitinase UCHL3 could deubiquitinate Rad51 at Lys56/57/63 and enhance the Rad51-BRCA2 interaction for proper HRR (16). Rad51 phosphorylation at Thr307/309 by CHK1 guarantee the binding of Rad51-BRCA2 and the subsequent recruitment of Rad51 to sites of DNA damage (17). Both transcriptional and post-translational modifications of Rad51 are essential for the expression level and function of Rad51, which may open new avenues for anti-tumor strategies.

**Table 1:** Regulators of RAD51 expression pattern.

Regulator	Function	Level	Effect
**CDK12**	transcription factor	mRNA	activator
**CDK13**	transcription factor	mRNA	activator
**E2F1**	transcription factor	mRNA	activator
**FOXM1**	transcription factor	mRNA	activator
**HMGA1**	transcription factor	mRNA	activator
**E2F7**	transcription factor	mRNA	repressor
**E2F4**	transcription factor	mRNA	repressor
**p53**	transcription factor	mRNA	repressor
**UCHL3**	de-ubiquitinase	protein	activator
**CHK1**	kinase	protein	activator

MicroRNAs (miRNAs) are small endogenous RNAs that mediate post-transcriptional regulation of gene expression, which have been reported to be dysregulated in a variety of cancers and regarded as therapeutic candidates. Multiple studies have shown that the functions of Rad51 are mediated by multiple dysregulated miRNAs. For instance, miR-155 binds with the 3’-untranslated region (3’-UTR) of Rad51 to regulate DNA repair capability and response to irradiation in breast cancer (18). In addition, miR-96 and directly targets the coding region of Rad51, and overexpression of miR-96 in tumor cells reduces the levels of Rad51 and sensitizes tumor cells to DNA damage agents (19). Besides, miR-182 has been identified to target Rad51 induced by HDAC inhibition, sensitizing acute myelocytic leukemia cells to DNA-damaging agents that activate HRR as a potential resistance mechanism (20). It has been found that Lnc-RI stabilizes Rad51 mRNA *via* competitive binding with miR-193a-3p to thus regulate HRR repair (21).

## Key Rad51 Mediators and Interactors Involved in Homologous Recombination

HRR signaling pathways can repair highly cytotoxic double-stranded DNA breaks and restore stalled replication forks (22, 23). Multiple Rad51 mediators and interactors participate in HRR of tumor cells. For instance, BRCA2 functions as a tumor suppressor that maintains chromosome integrity, and its deregulation by genetic mutations has been directly linked to tumorigenesis (24). It has been demonstrated that BRCA2 mediates the recruitment of Rad51 to DNA double-strand breaks to catalyze repair *via* HRR, making the BRCA2-Rad51 axis essential for HRR. During Rad51 nucleoprotein filament formation, BRCA2 primarily mediates Rad51 loading, which binds Rad51 through its BRC repeats and C-terminal domain. BRCA2 also interacts and coordinates with other mediators including DSS1 to enhance Rad51 loading. These observations provide a molecular basis for the role of BRCA2 in the maintenance of genome stability. The regulation of the BRCA2-Rad51 interaction has been extensively studied. For instance, UCHL3, a de-ubiquitinase phosphorylated and activated by ATM, could deubiquitinate Rad51 and enhance the interaction between Rad51 and BRCA2 for proper HRR (16). Besides, cyclin D1 could inhibit cyclin A-CDK2-dependent Ser3291 phosphorylation and facilitate Rad51 binding to the C-terminal domain of BRCA2, and downregulation of cyclin D1 leads to inefficient HRR (25). Early mitotic inhibitor 1 (EMI1), an F-box protein, assembles an active SCF ubiquitin ligase complex that constitutively targets Rad51 for proteasome-mediated degradation. Overexpression of Rad51 or depletion of EMI1 can bypass the need for BRCA1/2 to direct Rad51 to DNA double strand breaks, thereby making HRR functional (26) Cysteine-rich intestinal protein 1 (CRIP1), a member of the LIM/double-zinc finger protein family, promotes nuclear enrichment of Rad51. Upon DNA damage, CRIP1 is deubiquitinated and upregulated by activated AKT signaling, making CRIP1 as an essential target for regulating function of BRCA2-Rad51 axis (15). Trenner et al. identified a synthetic 16-mer peptide derived from the BRC4 repeat motif of BRCA2 is capable of blocking Rad51 binding to BRCA2, which may serve as a promising anticancer agent (27). Collectively, BRCA2-Rad51 axis plays a crucial role in the regulation of tumorigenesis (27).

RADX is another regulator of Rad51 that functions at replication forks to maintain genome stability (28). RADX regulates stalled fork reversal and protection by antagonizing Rad51 (4). Mechanistically, RADX competes with Rad51 for binding to single-stranded DNA, indicating that RADX buffers Rad51 to mediate fork protection to maintain genome stability (28). In addition to its single-stranded DNA binding ability, RADX interacts with Rad51 to maintain proper replication fork elongation rates and HR capacity. RADX can exert inhibitory and promoting effect for fork reversal according to replication stress levels, ensuring that replication functions of RAD51 are properly mediated (29). These findings indicate RADX as an essential mediator for proper Rad51 function and genome stability.

## Role of Rad51 in Tumorigenesis and Progression

### Rad51 and Tumor Metabolism

Oxidative stress, referring to overproduction of reactive oxygen species (ROS), has been indicated to be highly engaged in tumor initiation and development (30). Specifically, high levels of ROS could induce DNA damage and affect the DDR (31). In turn, nuclear DNA damage further induce mitochondrial response and promote the accumulation of mitochondrial ROS (mtROS) to further exacerbate nuclear DNA damage. In ovarian cancer, Rad51 depletion exhibits accumulation of mtROS and impaired mitochondrial membrane potential (32). Specifically, blockade of Rad51 can impair HRR to increase DNA damage and CHK1-dependent cell arrest at G2/M phase, which ultimately increases ROS accumulation to further enhance nuclear DNA damage. Given that ROS has been considered as a two-edged sword, the mutual effect between Rad51 and ROS should be extensively studied.

Although glycolysis is less efficient than oxidative phosphorylation in the production of ATP, tumor cells adapt to nutrient-deprived environment *via* increasing uptake of glucose to sustain high rates of glycolysis (33). Additionally, glycolysis also provides building materials for macromolecule synthesis, thus supporting survival of tumor cells. Therefore, targeting glycolysis pathway in cancers is a well-established therapeutic strategy. Recent study discovered that the combination therapy of glycolytic inhibitor 2-deoxy-D-Glucose and Rad51 specific inhibitor has shown increased efficacy for targeting leukemias, indicating increasing the efficacy of glycolytic blockade in tumor cells *via* Rad51 inhibition (34). Moreover, it has been found that Rad51 upregulates aerobic glycolysis by modulating HIF1α protein stability and HIF1α-targeted transcriptional program to mediate malignant behaviors of pancreatic tumor cells (35). With more and more evidence showing that Rad51 may participates in the metabolic adaptation of tumorigenesis, the role of Rad51 in tumor metabolism and its mechanisms require more studies. Specifically, the deeper mechanisms by which Rad51 regulate metabolic rewiring of tumor cells need to be studied, including how Rad51 regulates the expression of glycolytic proteins and activates specific downstream signaling pathways. Determining the mechanisms of how Rad51 mediates tumor adaptation brings promising strategies for cancer treatment.

### Rad51 and Tumor Metastasis

Metastasis is the major cause of cancer-related deaths (36). Epithelial-mesenchymal transition (EMT) is a cellular program defined as the transformation of epithelial cells into motile mesenchymal cells, which is critical for malignant progression (37). Moreover, EMT confers tumor cells enhanced tumor-initiating and metastatic potential. Increasing evidence indicated that genomic instability is essential for tumor metastasis (38). Thus, the role of Rad51 in the EMT program of tumor cells has also been studied. In esophageal squamous cell carcinoma, high Rad51 expression promotes tumor metastasis through the p38/Akt/Snail signaling pathway in TE8, CE81T, and KYSE70 cells (39). Additionally, EGFR-Erk1/2/Akt-Rad51 axis regulates EMT and DNA repair pathways in prostate cancer (10). SIM2s, a transcription factor from bHLH/PAS family, regulates DNA damage repair through enhancement of HRR, and prevents EMT in an ATM-dependent manner. SIM2s interacts with ATM and is stabilized through ATM-dependent phosphorylation in response to irradiation. Once stabilized, SIM2s interacts with BRCA1 and supports Rad51 recruitment to the site of DNA damage. Blockade of SIM2s can reduce HRR efficiency through disruption of Rad51 recruitment, resulting in genomic instability and induction of EMT (40). Therefore, Rad51 mediates tumor metastasis through multiple mechanisms, which may provide new therapeutic targets for overcoming tumor progression.

### Rad51 and Chemotherapeutic Resistance

Chemotherapeutic resistance is a key factor affecting the efficacy of therapeutic strategies in cancer treatment. The resistance of tumor cells to chemotherapeutic drugs, such as cisplatin, remains a major challenge to patient recovery. It has been found that BMI1-Rad51 axis is critical for reducing cisplatin-induced DNA damage. In breast cancer stem cells (bCSCs), BMI1 has been located to stalled replication forks to recruit Rad51 and activate HRR pathways, whereas BMI1 cannot activate HRR pathways in non-bCSCs (41). Moreover, Rad51 inhibition sensitizes stem cells to cisplatin. Collectively, BMI1-Rad51 axis mediates drug resistance of bCSCs to DNA-damaging agents and provides evidence that inhibiting Rad51 can chemosensitize bCSCs. C-terminal binding protein 1 (CtBP1), a transcription corepressor, confers breast tumor cells resistance to cisplatin by Rad51 upregulation in both breast cancer and gastric cancer cells (9, 42) . Rad51 expression and stability is critical for nucleolar and spindle-associated protein 1 (NUSAP1)-mediated chemoresistance *via* DDR signaling in chronic lymphocytic leukemia cells (43). Rad51 is also positively regulated by Jab1 to impair the therapeutic response to cisplatin-based chemotherapy, whereas Jab1 inhibition leads to impaired Rad51 expression for enhancing chemotherapeutic response (44). In epithelial ovarian cancer, high expression of Rad51 has been found to be correlated with early relapse after platinum-based regimens and impaired cytotoxic T cell infiltration (45). Therefore, Rad51 serves as a determinant of platinum resistance and a novel therapeutic target to overcome immune escape in Rad51-high epithelial ovarian cancer.

### Rad51 and Resistance to PARP Inhibitors

Defective HRR not only enhances sensitivity of germline BRCA-mutated tumors to chemotherapeutic agents, but also to PARP inhibitors that impair DNA repair pathways. In the clinical settings, PARP inhibitors such as olaparib and rucaparib have been approved for the indications of metastatic breast cancer and patients with recurrent ovarian cancer with disruptive mutations in BRCA1/2, showing well-tolerated trait and anticancer efficacy (46). It has been well-established that Rad51 functions as a promising predictor for the identification of PARP inhibitor-sensitive tumors in multiple tumor types (47). Basal Rad51 foci score acts as a candidate predictive biomarker of olaparib response in ovarian cancer patient-derived xenografts (48). Cruz et al. found that low Rad51 expression was correlated with objective response to PARP inhibitors in germline BRCA-mutated tumors, indicating Rad51 as a valuable biomarker to select patients eligible for treatment of PARP inhibitors (49).

The underlying mechanisms for resistance of PARP inhibitors are complex and extensively studied, and combination therapy may provide new avenues to overcome resistance to PARP inhibitors. Cancer stem cells (CSC) are considered to drive this resistance to PARP inhibitors. In BRCA1-mutant TNBCs, CSCs are resistant to PARP inhibitors, and that these cells display elevated Rad51 protein levels and foci formation. (8). Considering that the contribution of enhanced DNA repair capacities to resistance of PARP inhibitors, combination treatment of PARP inhibitors and Rad51 inhibitors may be a promising option to improve therapeutic response of PARP inhibitors. The negative elongation factor (NELF), a four-subunit protein complex, has been recognized as a new component in the DNA damage response to mediate repair of double-strand break (50). In hepatocellular carcinoma, NELF complex mediates BRCA1 and Rad51 recruitment to DNA damage sites and therefore regulates sensitivity to PARP inhibitors (51). Thus, NELF-E inhibition can sensitize Hep3B cells to PARP inhibitors by impairing the recruitment of Rad51 to the DNA damaging sites. Collectively, combination therapy may open new avenues for overcoming resistance to PARP inhibitors.

## Clinical Implications of Rad51 in Cancer

### Prognostic and Predictive Value of Rad51 in Cancer

Currently, genetic examinations measuring homologous recombination deficiency (HRD) exhibit limited predictive value (52). Therefore, predictive molecular target for the status of HRD is urgently needed. Worth noticing, Rad51 test has been found to identify tumors with HRD and is highly concordant with genomic HRD. Tumors with HRD mutational signatures harboring a functional defect in HR can be evaluated by reduced Rad51 foci formation (53). Moreover, Rad51 independently predicts clinical benefit from adding carboplatin to neoadjuvant chemotherapy (NAC) in triple-negative breast cancer. In patients received NAC, the rates of completed pathological responses are higher in Rad51-negative case (54). Thus, baseline Rad51 expression can serve as a predictive factor for the response to NAC.

Dysregulated expression of Rad51 has been commonly discovered in various tumor types. Moreover, dysregulated expression of Rad51 is associated with diverse clinic-pathological factors and prognosis, indicating Rad51 as a potential prognostic marker in many tumor types. For instance, Rad51 expression in esophageal squamous cell carcinoma was associated with advanced lymph node metastasis and unfavorable survival outcomes (55). In glioblastoma, Rad51 was overexpressed and negatively associated with overall survival (5). Herein, Rad51 expression may serve as promising prognostic factor in the clinical settings.

### Pharmaceutic Inhibitor of Rad51 in Cancer

For the past decade, Rad51 has been regarded as a promising therapeutic target for novel therapeutic inhibitors. Rad51 inhibitors may sensitize tumor cells to chemotherapeutic agents, render tumors to be more efficient in HRR, and to be more responsive to PARP inhibitors targeting HRR-deficient tumors with mutated BRCA1/2 genes. 4,4’-diisothiocyanato-stilbene-2,2’-disulfonic acid (DIDS) molecule is a newly identified Rad 51 inhibitor (56, 57). DIDS and its two analogs can prevent Rad51 binding to single-stranded DNA and Rad51-mediated D-loop formation to HRR function. Another Rad51 inhibitor B02 was recently discovered to induce HRR deficiency in TNBC, sensitizing MDA-MB-231 cells to the PARP inhibitor (58). It has been demonstrated that B02 could reduce DNA DSB repair and lead to radio-sensitization in glioblastoma stem cells, indicating Rad51 as a crucial and selective DNA repair target for tumor stemness (59). IBR2 is a recognized Rad51 inhibitor to impair Rad51 multimerization and promote proteasome-mediated degradation of Rad51 protein to therefore reduce HRR function, which ultimately enhances apoptosis and impairs tumor growth of chronic myeloid leukemia (60). In addition to directly targeting Rad51 protein, destruction of the protein-protein interaction between BRCA2 and Rad51 can also impair HRR and mediate cell death for development of anti-tumor strategies. Besides, Rad51 promoter-based anticancer therapy may also function as a promising therapeutic strategy. It has been found that the fusion of Rad51 promoter to diphtheria toxin A gene impair the initiation of multiple tumor types, such as breast and cervical tumor, with minimal effect on normal epithelial cells (61, 62). Thus, therapies based on the Rad51 promoter will be highly tumor-specific and open new avenues for targeting a variety of tumor types.

## Conclusions

In conclusion, Rad51 has been observed to be dysregulated in various tumor types, and associated with unfavorable clinicopathological factors and prognosis. Rad51 serves as a key regulator for proper HRR to ensure DNA repair. Besides, Rad51 also participates in the tumor metabolism, metastasis and chemotherapeutic resistance. Rad51 functions as a promising predictor for the identification of tumor eligible for the treatment of PARP inhibitor. Multiple Rad51 inhibitors have been developed to be utilized to overcome chemotherapeutic agents and PARP inhibitors. Collectively, Rad51 is a promising therapeutic target for developing anti-tumor strategies, waiting for deeper investigation.

## Author Contributions

ZW: Conceptualization and Writing of the first draft. RJ, LW, QY, XH, QF, XZ: Review and editing. WL, YR: Conceptualization and Review. All authors contributed to the article and approved the submitted version.

## Conflict of Interest

The authors declare that the research was conducted in the absence of any commercial or financial relationships that could be construed as a potential conflict of interest.

## Publisher’s Note

All claims expressed in this article are solely those of the authors and do not necessarily represent those of their affiliated organizations, or those of the publisher, the editors and the reviewers. Any product that may be evaluated in this article, or claim that may be made by its manufacturer, is not guaranteed or endorsed by the publisher.
